# Complications of Laparoscopic Hysterectomy: Avoid the Risks

**DOI:** 10.1155/DTE.3.9

**Published:** 1996

**Authors:** James E. Carter

**Affiliations:** Advanced Surgical Education Associates Women's Health Center of South Orange County 26732 Crown Valley #541 Mission Viejo CA 92691 USA

## Abstract

Results of more than 1500 laparoscopic hysterectomies have been reported since 1989. The complication rate is 10 to 11% in expert hands. However, a complication rate of 60% was reported in one community hospital series. The risks of laparoscopic hysterectomy are acceptable at 10% but not at 60%. Practitioners of laparoscopic assisted vaginal hysterectomy must review and understand the risks associated with the procedure in order to avoid them. This article addresses the complications of laparoscopic hysterectomy and gives techniques to avoid them.


*Diagnostic and Therapeutic Endoscopy*, Vol. 3, pp. 9–17
Reprints available directly from the publisher
Photocopying permitted by license only

© 1996 OPA (Overseas Publishers Association)
Amsterdam B.V. Published in The Netherlands
by Harwood Academic Publishers GmbH
Printed in Singapore

### Complications of Laparoscopic Hysterectomy: Avoid the Risks

JAMES E. CARTER*,†

*Advanced Surgical Education Associates, Women's Health Center of South Orange County, 26732 Crown Valley #541, Mission Viejo, CA 92691*

*(Received 2 June 1995; In final form 22 March 1996)*

**Results of more than 1500 laparoscopic hysterectomies have been reported since 1989. The complication rate is 10 to 11% in expert hands. However, a complication rate of 60% was reported in one community hospital series. The risks of laparoscopic hysterectomy are acceptable at 10% but not at 60%. Practitioners of laparoscopic assisted vaginal hysterectomy must review and understand the risks associated with the procedure in order to avoid them. This article addresses the complications of laparoscopic hysterectomy and gives techniques to avoid them.**

*Keywords:* Laparoscopy, hysterectomy, complications

### COMPLICATIONS OF LAPAROSCOPIC HYSTERECTOMY—REVIEW

The complications of a surgical procedure can be identified only through a review of a large number of completed procedures. It is helpful to the understanding of the complications that can occur with laparoscopic assisted vaginal hysterectomy (LAVH) to summarize the reports now available in the literature on complications that have occurred with this procedure. In the first 67 LAVH procedures performed by Carter and Bailey [1], there were two instances of vaginal cuff cellulitis and one pulmonary infection requiring intravenous antibiotics. There were also one Richter's hernia, one lacerated inferior epigastric vessel, and two urinary tract infections in this series for a 10.5% complication rate. After introduction of the use of prophylactic antibiotics including cefotetan disodium and metronidazole hydrochloride, there have been no further cases of cuff cellulitis in the subsequent 200 procedures. Pulmonary infections have been avoided by the use of postoperative incentive spirometry started immediately upon the patient's awakening. Richter's hernia is avoided by using the Carter-Thomason fascial closure device in a mass closure of all 10 mm and greater port sites [2].

Hill *et al.* [3] reported a 15.9% complication rate in the first 200 LAVH procedures. Anterior abdominal wall vessel injury occurred in 5 patients, bladder injury in 5, febrile illness in 13, secondary hemorrhage in 4, Richter's hernia in 1, and temporary ureteral obstruction in 4. Five of the patients with febrile

*Corresponding author. Tel.: 714 3645802. Fax: 714 3642821.

episodes actually had pelvic hematomas. One required general anesthesia to evacuate the hematoma. In the study of Padial *et al.* [4] of 75 patients undergoing LAVH, there was a 10.6% frequency of febrile morbidity with no organ injuries or wound infections with a mean hospital stay of 2.4 days.

In 839 LAVH procedures reviewed by Ou *et al.* [5], there were 8.8% minor complications and 2.7% major complications. The minor complications included urinary tract infections in 2%, fever in 1.2%, hematoma in 1.2%, hemorrhage in 1.1%, anemia in 1%, hematuria in 0.6%, ileus in 0.5%, nausea in 0.4%, urine retention in 0.25%, abdominal pain in 0.25%, and arrhythmia, knee pain, tendonitis, and leg numbness in 0.1%. Major complications included 8 bladder injuries (1%), hemorrhage (0.9%), hernia (0.4%), trocar injury to epigastric vessels (0.35%), and pulmonary embolus 2 weeks postoperatively (0.1%).

Liu and Reich [6] reported on the overall complication rate in 518 total laparoscopic hysterectomies of 5.76% with 2.12% of patients having febrile morbidity, which included pneumonia, pelvic hematoma, dehydration, and transient febrile episodes. The only death associated with LAVH was reported in this series. The patient developed bilateral pneumonia with adult respiratory distress syndrome after her discharge from her initial operation. Boike *et al.* [7] reported two bowel obstructions in 82 LAVH procedures, both occurring as a result of bowel herniation into port sites of 10 mm and greater. Woodland [8] reported on transection and ligation of the ureter with the linear stapling device. Kadar and Lemmerling [9] reported on ligation of the ureter with suture during the vaginal portion of the procedure. Bernstein *et al.* [10] pointed out that the complication rate with LAVH drops with the experience of the operators and that the rates can be comparable with vaginal hysterectomy even in a community hospital [11].

Dicker *et al.* [12] demonstrated conclusively that complications of vaginal hysterectomy are far fewer than those for abdominal hysterectomy. Dicker *et al.* [13] also demonstrated the trend toward abdominal hysterectomy in spite of the advantages of the vaginal approach. Nezhat *et al.* [14] and Carter *et al.* [15] clearly demonstrated that LAVH patients require fewer days of hospitalization, return to work much sooner, and require far less pain medicine than abdominal hysterectomy patients.

Summit *et al.* [16] demonstrated that LAVH had no advantage over vaginal hysterectomy in patients in whom vaginal hysterectomy could be performed. By using gonadotropin releasing hormone (GnRH) agonist for 2 months before hysterectomy, Stovall *et al.* [17] were able to reduce uterine size by an average of 47%. Of patients given GnRH agonists, 80% had vaginal hysterectomies while 75% of patients who had not received GnRH agonists had abdominal hysterectomies. Techniques such as those described by Groty [18] for extracting the large uterus were used extensively. Pelosi and Kadar [19] have demonstrated that even very large uteri (≥500 g) can be safely removed vaginally by combining the laparoscopic approach with the principles described by Groty [18].

Galen *et al.* [20] have demonstrated that with proper patient preparation and selection the majority of LAVHs can be performed in an outpatient setting. Lyons [21] demonstrated that the supracervical approach to the laparoscopic hysterectomy is even safer than the LAVH in his comparative study. Since Reich *et al.* [22] first reported on the LAVH in 1989, safe introduction of the technique into the practice of skilled surgeons has been extensively reported [23–30].

However, Schwartz [31] pointed out that the complication rate can be unacceptably high (50%) when the procedure is performed by surgeons not sufficiently trained in these procedures. Shwadyer [32] has demonstrated that there is a significant learning curve for the procedure, which results in a decreasing complication rate. Kadar [33] developed an elegant and reproducible technique to avoid complications with the laparoscopic hysterectomy procedure, which, however, requires a retroperitoneal approach to the hysterectomy. Smith *et al.* [34] in a landmark study reported a 60% complication rate in LAVH procedures at a community hospital. Excess blood loss occurred in 8 patients (mean 767 ml) and cystotomy in 1.

As the transfer of the technology associated with LAVH takes place from the very skilled laparoscopists to the gynecologists who are practicing laparoscopy as a part of their general practice, complication rates appear to increase significantly. To eliminate certain complications (Richter's hernia) and to reduce significantly the likelihood of others (cuff infection, pulmonary infection, epigastric vessel laceration, and ureter injury) certain principles for the performance of this procedure have been developed.

### PERFORMANCE OF THE PROCEDURE

### Patient Selection

The key to a successful outcome for a laparoscopic hysterectomy is proper patient selection. Patients who cannot undergo appropriate anesthesia or who have a high risk for laparoscopic procedures should not be counseled to undergo these procedures. Topel's technique of gasless LAVH under epidural anesthesia [30] allows for the extension of this procedure to those who cannot tolerate general anesthesia.

Patients should be counseled for the possibility of open exploratory surgery. Although in certain hands, a large uterus can be safely removed [19], certainly the initial procedures of laparoscopists should not involve uteri 500 g.

LAVH should be avoided in patients with extensive adhesions of the bowel, and general surgical consultation should be obtained if these are found unexpectedly and require dissection. Extensive stage IV endometriosis that involves either the ureters or the bowel or that is extensive enough to form an obliterated cul-de-sac, requires great care in dissection and may in fact obligate the surgeon to abandon the laparoscopic approach and perform an open procedure (31).

The golden rule of laparoscopic surgery is, "Know thy limits." What is appropriate and appears easy for the highly skilled laparoscopist may in fact lead to disaster for a person of average skill. More than any other form of surgery, laparoscopic surgery depends on certain inherent qualities of eye-hand coordination and the ability to operate in a closed environment using two-dimensional images. Inherent talents may vary considerably from one surgeon to another [31].

### Patient Preparation

Patients in whom any bowel adhesions or bowel disease is suspected should be given mechanical osmotic preparation solutions for bowel cleansing preoperatively. In addition, preoperative antibiotics with a regimen such as 1 g of cefotetan disodium and 500 mg of metronidazole hydrochloride is appropriate. With this combination of antibiotics, if the bowel is entered it can be safely repaired by primary closure performed laparoscopically. Also, pneumatic compression stockings should be used to reduce the risk of venous thrombosis and pulmonary embolus.

The anesthetist should empty the stomach with an orogastric or nasogastric tube and should avoid the use of nitrous oxide. This will reduce the risk of Verres needle or trocar injury to the transverse colon. A Foley catheter should be placed to ensure that the bladder has been completely emptied to avoid trauma to the bladder during placement of the secondary trocars.

### Patient Positioning

The patient should be positioned on a mechanical table which allows sufficient tilt for a steep Trendelenberg position and should have the legs appropriately placed to allow the performance of the vaginal part of the procedure. Great care must be taken to avoid pressure on the peroneal nerve as well as excess traction on the femoral or sciatic nerve to ensure that no nerve palsies result. The arms should be placed at the sides to avoid excess traction in the area of the brachial plexus, and the surgeon should be provided with adequate space by having the anesthetist posistion the anesthesia equipment well up at the head of the table with no excess equipment in the surgeon's way. The surgeon may prefer to begin with the patient in a modified lithotomy position with the thighs nearly horizontal and the knees flexed (Fig. 1) and then elevate the knees and feet to provide room to complete the vaginal portion of the procedure. The author has found it possible to place the legs in a position 60° from horizontal and 30° from vertical, which allows the author to perform the abdominal portion of the procedure without interferance from the extremities and then procede to

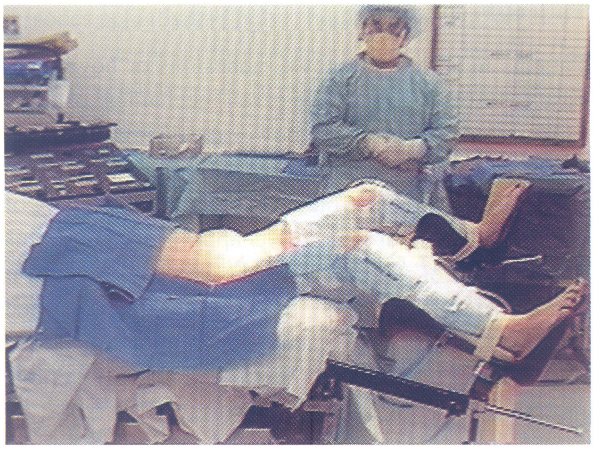

FIGURE 1 Patient is placed in dorsal horizontal position with appropriate stirrups as shown.

the vaginal portion of the procedure without the need for a change in the positions of the stirrups (Fig. 2).

### Trocar Placement and Position

Introduction of the trocar can be performed either in a closed or an open procedure and can be preceded or not preceded by insufflation through a Verres' needle [35]. The most important principle to follow for the introduction of the trocars is to avoid areas where adhesions are likely. Many surgeons will simply use the umbilical incision point regardless of whether previous surgery has taken place. However, studies indicate that as many as 35% of patients with previous vertical incisions and 25% with Pfannenstiel's incisions will have adhesions extensive enough near the umbilicus that these create dangers for the individual patient [36]. In these patients, the use of the Palmer [37] point (Figs. 3 and 4) (midclavicular line, left upper quadrant) is more appropriate for the placement of the initial trocar. The author uses the Palmer point in all cases where adhesions are suspected because of previous surgery. For placement of the initial trocar at Palmer's point, the author prefers to use a 5-mm trocar with direct puncture while elevating the abdominal wall just below the point of entry. The trocar is inserted in a near vertical plane oriented only a few degrees toward the caudad direction. If the Verres' needle is used, its position should be verified with the drop test or injection of 20 ml of normal saline and withdrawal on the syringe. At withdrawal on the syringe there should be an aspiration feeling of a vacuum as the properly placed needle will be inside the peritoneal cavity, which has a negative pressure. After the position check, the $CO_2$ insufflator can be attached and the insufflation initiated at low rates (1 liter/min) and the initial pressure of insufflation carefully checked. This should be less than 6 mm Hg [15].

After insufflation to 15 mm Hg pressure, the initial trocar placement can be performed. Perhaps the safest way to approach this is to place a small 5 mm trocar at the umbilicus, check the position with a 5-mm laparoscope, and then dilate the trocar site with an appropriate dilating system to allow for placement of the diagnostic 10-mm scope. A second approach is to simply place the 10-mm trocar to allow for the insertion of diagnostic 10-mm scope.

Trocar placement is an operator preference, but if 12-mm trocars are used for a linear stapling device, the entry point should be chosen well lateral to the inferior epigastric vessels on either side and lateral as well to the rectus sheath. Care must be taken to identify the position of the superficial circumflex artery and vein. All these vessels should be avoided, and yet at times, bleeding will still occur whether with a 5- or a 12-mm puncture. When this occurs, the bleeding can be stopped by placement of sutures appropriately introduced by one of the available emergency needles or suture passers currently present in most operating rooms [2]. Trocar placement should be adjusted for the body habitus of the patient and the size of the uterus, as well as the presence or absence of other diseases such as ovarian cysts, adhesions, or endometriosis.

Performance of the procedure can be accomplished with stapling devices, sutures, or cautery. All have certain inherent risks and advantages and disadvantages. Perhaps the most important concern with the use of the stapler is to ensure that the course of the ureter is well-demarcated and to ensure that no bleeding occurs from the ligated stapled pedicles. If bleeding is noted, this should be treated with the careful application of bipolar cautery in the area behind the staple line. If bipolar cautery is used, again the sur-

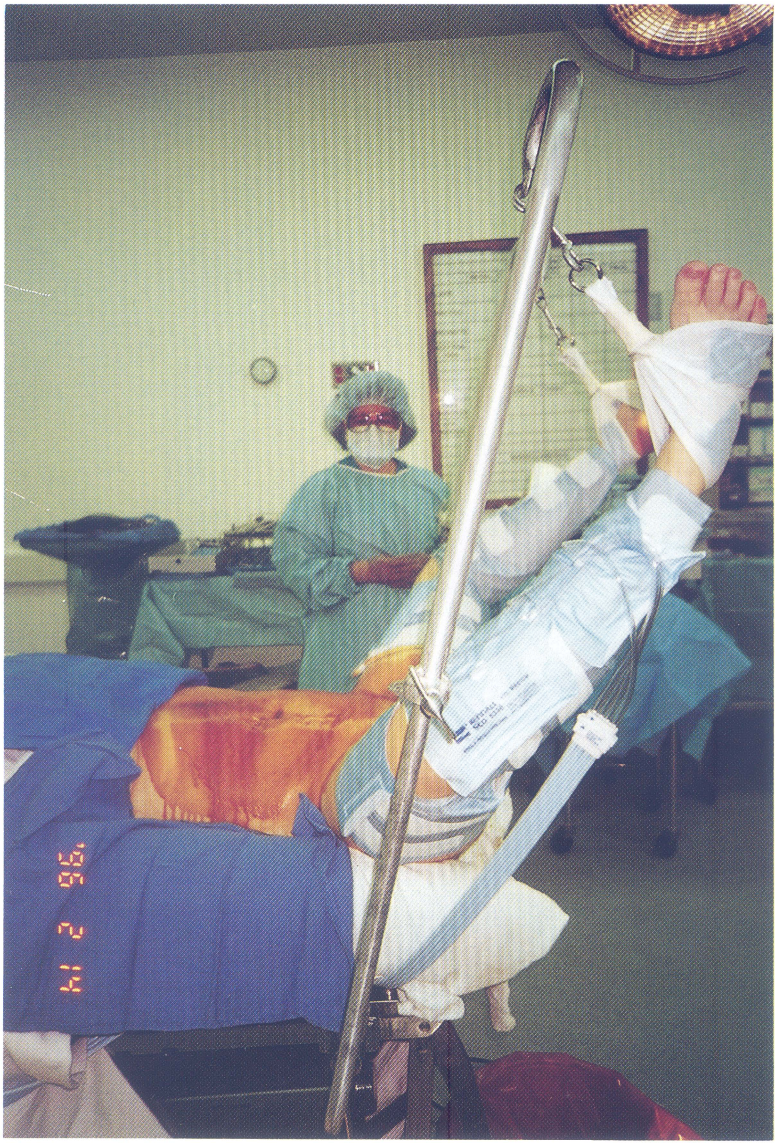

FIGURE 2 Leg position in stirrups so that no change in position is required to change from laparoscopic to vaginal position.

geon must be aware of the possibility of thermal transfer of energy, because an application of 8 sec of bipolar energy in the area of the uterine artery can lead to a increase in temperature 1 cm away from the point of application that is approximately 100°C and exceeds the temperature at which tissue can remain viable [38]. Care must be taken to apply the bipolar coagulation instrument only to the point when boiling ceases and avoid the point of carbonization so that a well-sealed vessel is obtained. Suture techniques are certainly appropriate and relatively straightforward for the surgeon. With the development of new suture devices, the possibility exists that suturing will become simple enough for the average gynecologic laparoscopist. At the present time and with current instrumentation, suturing is time-consuming and can lead to significant frustration for the laparoscopist. However, suturing skills should be mastered so that appropriate suturing

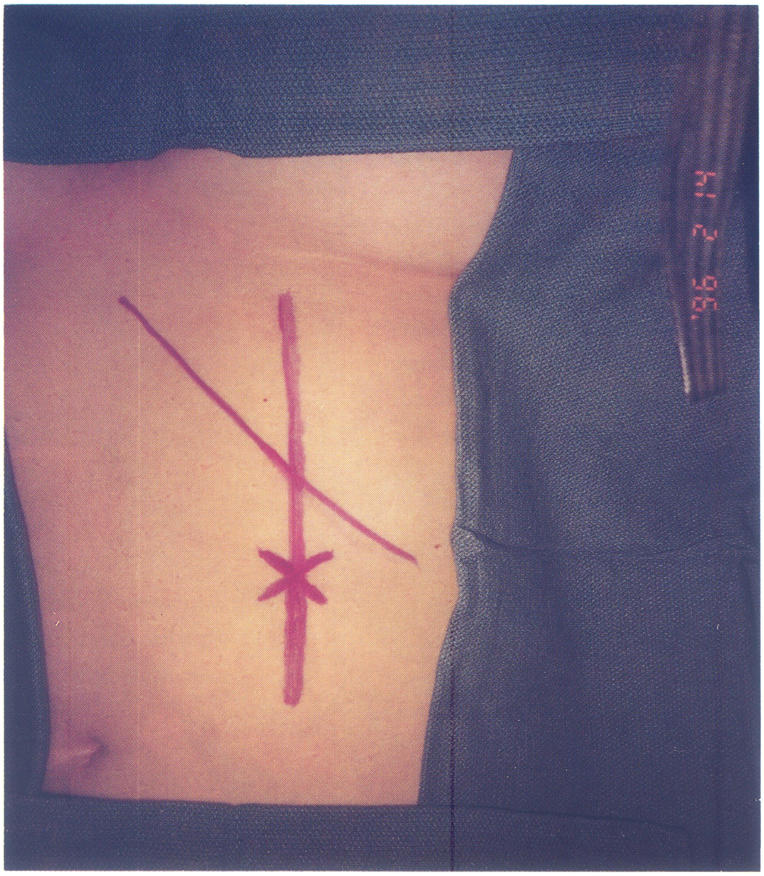

FIGURE 3 Location of Palmer's print.

procedures can be performed, such as simple repair of small lacerations of the bladder.

### Completion of the Procedure

The laparoscopic hysterectomy can be completed either laparoscopically or vaginally, and either a supracervical or total hysterectomy can be performed. The surgeon must remember that if the procedure is completed vaginally, care must be taken to keep all clamps very near the cervix and uterus to ensure that the ureter is not injured on this portion of the surgery. Especially at the point of taking the angle sutures for cuff closure, the surgeon must avoid too deep a placement in lateral position because of the course of the ureter at this point.

For large uteri, morcellation and bivalving techniques should be studied and utilized [18]. It helps to pretreat the large uterus with GnRH agonist to reduce its size so that it is more easily and readily removed at the point where the vaginal approach is undertaken [17].

### Final Look and Exiting from the Abdominal Cavity

After complete closure, irrigation is used to carefully evaluate all pedicles and potential areas of bleeding. In addition, the bowel is carefully inspected for injury. Before instruments are removed, 20 ml of 0.25% bupivacaine can be injected into the pelvic cavity to help provide for pain relief. The instruments and trocars are removed one by one, and as each trocar is removed, the incision site for that trocar is closed using through

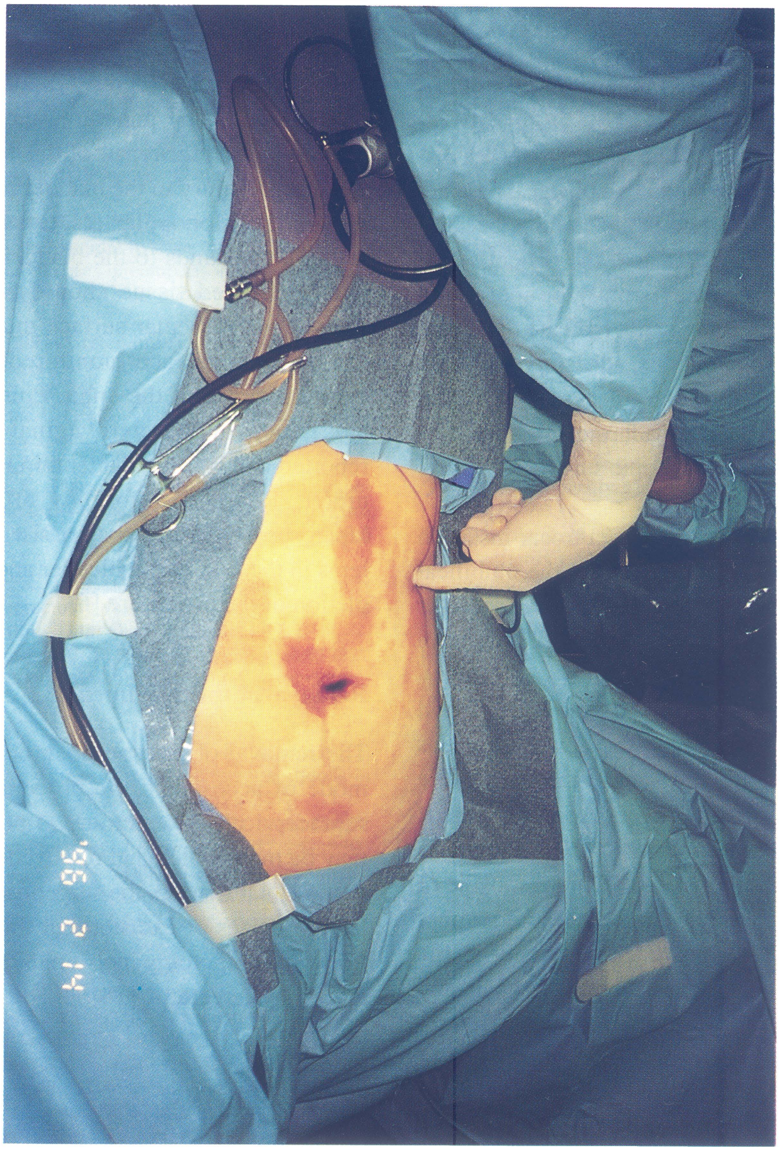

FIGURE 4 Finger points to Palmer's Point.

and through mass closure techniques to ensure that muscle, fascia, and peritoneum are incorporated into the closure. Techniques for doing this have been described and instruments are available to ensure that this closure is complete to avoid problems with incisional hernias [2]. Instruments are removed under direct vision, including the final removal of the umbilical trocar and the laparoscope.

All incision sites are injected with 0.25% bupivacaine. The anesthetist should keep the patient at normal temperature with the use of heating aids and by ensuring that appropriately warm solutions are used during the surgery. The patient should be evaluated postoperatively with a hemoglobin check to ensure that no postoperative bleeding is occurring. Patients can be discharged home once they are ambulatory and have active bowel sounds.

### DISCUSSION

With all of these precautions, complications still can and do occur.

First, there can be complications associated with dissection of both small bowel and large bowel. These are the most dangerous of all the complications because damage is frequently unrecognized; the patient's presentation postoperatively may simply be increasing pain and laboratory studies may not indicate the extent of the damage [39]. To avoid this, adhesiolysis should only be undertaken if absolutely necessary for pain relief and visualization, and the adhesiolysis should be performed by an expert laparoscopic surgeon to ensure minimal likelihood of damage.

Bowel can also be injured by direct trocar insertion and by Verres' needle insertion. Avoid the umbilical site for entry in patients with previous vertical or Pfannenstiel's incisions, as well as in those who have had previous umbilical surgery [36,40].

Recognize that thermal injury to the bowel could occur, especially in areas where the bowel underlies the secondary trocar sites and is not visualized while surgery is being performed. This is especially true if a monopolar insulated electrosurgical instrument is placed within a conducting sheath, which is inside of an insulating threaded anchoring device. Remember that a white blanched appearance occurring in the area of the bowel indicates deep thermal damage, and a black charred area indicates more superficial damage. Bowel can also be injured in the process of resecting endometriosis, and if any possibility of damage exists, examination by insertion of a rigid sigmoidoscope and insufflation of the bowel under water can be performed [39]. Bowel can also be injured at the end of the procedure by leaving a peritoneal or fascial defect and having the bowel work its way into this defect. The complete closure of all fascial incisions >5 mm, including closure of fascia, muscle, and peritoneum, will reduce this complication rate significantly [2].

Ureteric complications can occur if the position of the ureter has not been carefully identified, or if the ureter is misidentified in the process of either suturing or coagulation or stapling. Placement of ureteric stents and the use of ureteric stents with lighting capability can be helpful in difficult procedures in which the ureter is difficult to identify [8]. The bladder itself can be traumatized by puncture, and skill in suturing is most important for repair of this type of lesion [3].

Thermal injury to the bladder requires careful evaluation [3]. Monopolar electrosurgery can result in deep burn wounds to the bladder, and if such occur, the devitalized tissue should be resected and the serosal and muscular surface must be oversewn and the mucosal surface monitored for up to 3 weeks before removal of the drain. Episodes of vesicovaginal fistula and ureterovaginal fistula have been reported and increased attention is appropriate for the ureter. Routine cystoscopy after performance of laparoscopic surgery may assist in identifying problems in the area of the bladder and ureter before completion of the procedure [6].

### Removal of the Specimen

The removal of the specimen is dependent upon the mode of access and the procedure performed. If a supracervical hysterectomy is performed, the uterus may be morcellated and removed out through a trocar site [21]. Otherwise, the surgeon must rely on morcellating devices or excision out through the vagina, or through a colpotomy incision in the event of a supracervical hysterectomy [18].

### CONCLUSIONS

Following the recommendations listed here will not prevent the occurrence of laparoscopic complications, but should reduce their frequency.

### *References*

[1] Carter, J. E., Bailey, T. S. (1994). Laparoscopic assisted vaginal hysterectomy utilizing the contact tip Nd:YAG laser: a review of 67 cases, *Ann Acad Med Singapore*, **23**, 13–17.
[2] Carter, J. E. (1994). A new technique of fascial closure for laparoscopic incisions, *J Laparoendosc Surg*, **4**, 143–147.
[3] Hill, D. J., Maher, P. J., Wood, C. E., *et al.* (1994). Complications of laparscopic hysterectomy, *J Am Assoc Gynecol Laparosc*, **1**, 159–162.

[4] Padial, J. G., Sotolongo, K., Casey, N. J., *et al.* (1992). Laparos-copically assisted vaginal hysterectomy. Report of 75 consecutive cases, *J Gynecol Surg*, **8**, 81–85.
[5] Ou, C. S., Beadle, E., Presthus, J., *et al.* (1994). A multicenter review of 839 laparoscopic vaginal hysterectomies, *J Am Assoc Gynecol Laparosc*, **4**, 417–422.
[6] Liu, C. Y., Reich, H. (1994). Complications of total laparoscopic hysterectomy in 518 cases, *Gynaecol Endosc*, **3**, 203–208.
[7] Boike, G. M., Elfstrand, E. P., Delpriore, G., *et al.* (1993). Laparoscopically assisted vaginal hysterectomy in the university hospital: report of 82 cases in comparison with abdominal and vaginal hysterectomy, *Am J Obstet Gynecol*, **168**, 1690–1701.
[8] Woodland, M. B. (1992).Ureter injury during laparoscopy assisted vaginal hysterectomy with endoscopic linear stapler, *Am J Obstet Gynecol*, **167**, 756–757.
[9] Kadar, N., Lemmerling, L. (1992). Urinary tract injuries during laparoscopically assisted hysterectomy: causes and prevention, *Am J Obstet Gynecol*, **170**, 47–48.
[10] Bernstein, P., Walla, K., Platt, L. D. (1994). Continuing experience with laparoscopically assisted vaginal hysterectomy in a private teaching community hospital, *J Am Assoc Gynecol Laparosc*, **1**, 53.
[11] Bernstein, P., Walla, K., Platt, L. D. (1994). Laparoscopically assisted vaginal hysterectomy versus vaginal hysterectomy in a private hospital setting, *J Am Gynecol Laparosc*, **1**, 53.
[12] Dicker, R. C., Greenspan, J. R., Strauss, L. T., *et al.* (1982). Complications of the abdominal and vaginal hysterectomy among women of reproductive age in the United States, *Am J Obstet Gynecol*, **144**, 841–848.
[13] Dicker, R. C., Scally, M. J., Greenspan, J. R., *et al.* (1982). Hysterectomy among women of reproductive age: trends in the United States, 1970–1978, *JAMA*, **248**, 323–327.
[14] Nezhat, F., Nezhat, C., Gordon, S., *et al.* (1992). Laparoscopic versus abdominal hysterectomy, *J Reprod Med*, **37**, 247–250.
[15] Carter, J. E., Ryoo, J., Katz, A. (1994). Laparoscopic assisted vaginal hysterectomy: a case control comparative study with total abdominal hysterectomy, *J Am Assoc Gynecol Laparosc*, **1**, 116–121.
[16] Summit, R. L., Stoval, T. T., Lipscomb, G. H., *et al.* (1992). Randomized comparison of laparoscopy assisted vaginal hysterectomy versus standard vaginal hysterectomy in an outpatient setting, *Obstet Gynecol*, **80**, 895–901.
[17] Stoval, T. G., Summit, R. L., Washburn, S. A., *et al.* (1994). Gonadatropin releasing hormone agonist use before hysterectomy, *Am J Obstet Gynecol*, **178**, 1744–1751.
[18] Groty, H. T. (1989). Vaginal hysterectomy: the large uterus, *J Gynecol Surg*, **5**, 301–312.
[19] Pelosi, M. A., Kadar, N. (1994). Laparoscopically assisted hysterectomy for uteri weighing 500 g or more, *J Am Assoc Gynecol Laparosc*, **1**, 405–409.
[20] Galen, D. I., Jacobson, A., Weckstein, LN. (1994). Outpatient laparoscopic assisted vaginal hysterectomy, *J Am Assoc Gynecol Laparosc*, **1**, 241–245.
[21] Lyons T. (1993). Supracervical laparoscopic hysterectomy. A comparison: morbidity and mortality results with LAVH, *J Reprod Med*, **38**, 763–767.
[22] Reich, H., DeCaprio, J., McGlynn, F. (1989). Laparoscopic hysterectomy, *J Gynecol Surg*, **5**, 213–215.
[23] Burke, R. K. (1994). Laparoscopic assisted vaginal hysterectomy in a community hospital: an initial experience, *J Am Assoc Gynecol Laparosc*, **1**, 59.
[24] Padial, J. G., Sotolongo, J., Ferrer, N. E. (1994). Our three year experience with laparoscopic assisted vaginal hysterectomy, *J Am Assoc Gynecol Laparosc*, **1**, 59.
[25] Hasson, H. M., Rotman, C., Rana, N., *et al.* (1993). Experience with laparoscopic hysterectomy, *J Am Assoc Gynecol Laparosc*, **1**, 1–11.
[26] Langebrekke, A., Skar, O. J., Urnes, A. (1992). Laparoscopic hysterectomy initial experience, *Acta Obstet Gynecol Scand*, **71**, 226–229.
[27] Maher, P. J., Wood, E. C., Hill, D. J., *et al.* (1982). Laparoscopically assisted hysterectomy, *Med J Aust*, **156**, 316–318.
[28] Minelli, L., Angeiolillo, M., Caione, C., *et al.* (1991). Laparoscopically assisted vaginal hysterectomy, *Endoscopy*, **23**, 64–66.
[29] Woodland, M. B. (1994). Laparoscopic assisted vaginal hysterectomy LAVH: data from 253 cases, *J Am Assoc Gynecol Laparosc*, **1**, 59.
[30] Topel, H. C. (1994). Gasless laparoscopic assisted hysterectomy with epidural anesthesia, *J Am Assoc Gynecol Laparosc*, **1**, 59.
[31] Schwartz, R. O. (1993). Complications of laparoscopic hysterectomy, *Obstet Gynecol*, **81**, 1022–1024.
[32] Shwadyer, J. M. (1994). The learning curve for laparoscopically assisted vaginal hysterectomy/laparoscopic hysterectomy, *J Am Assoc Gynecol Laparosc*, **1**, 533.
[33] Kadar, N. (1994). An operative technique for laparoscopic hysterectomy using a retroperitoneal approach, *Jl Am Assoc Gyecol Laparosc*, **1**, 365–377.
[34] Smith, D. C., Donohue, L. R, Waszae, S. J. (1994). A hospital review of advanced gynecologic endoscopic procedures, *Am J Obstet Gynecol*, **170**, 1635–1642.
[35] Dabirashrafi, H., Mohammad, K., Tabrizi, M. M., *et al.* (1994). The use of Verres' needle and 10 mm trocar vs. direct trocar insertion in the beginning of laparoscopy, *Jl Am Assoc Gynecol Laparosc*, **1**, 59.
[36] Levrant, S. G., Bieber, E., Barnes, R. (1994). Risks of anterior abdominal wall adhesions increase with number and type of previous laparotomy, *Jl Am Assoc Gynecol Laparosc*, **1**, 519.
[37] Chang, F. H., Lee, C. L., Soong, Y. K. (1994). Use of Palmer's point for insertion of the operative laparoscope in patients with severe pelvic adhesions: experience with 17 cases, *J Am Assoc Gynecol Laparosc*, **1**, 57.
[38] Phipps, J. H. (1994). Thermometry studies with bipolar diathermy during hysterectomy, *Gynecol Endosc*, **x**, 35–37.
[39] Soderstrom, R. M. (1993). Bowel injury litigation after laparoscopy, *J Am Assoc Gynecol Laparosc*, **1**, 74–77.
[40] Bieber, E. J., Levrant, S. (1994). The risk of anterior abdominal wall adhesions in patients with previous umbilical hernia repair, *Jl Am Assoc Gynecol Laparosc*, **1**, 54.

